# Prognostic values of geriatric nutritional risk index (GNRI) and prognostic nutritional index (PNI) in elderly patients with Diffuse Large B-Cell Lymphoma

**DOI:** 10.7150/jca.62340

**Published:** 2021-10-11

**Authors:** Dongmei Yan, Ziyuan Shen, Shuo Zhang, Lingling Hu, Qian Sun, Kailin Xu, Yingliang Jin, Wei Sang

**Affiliations:** 1 Department of Hematology, Affiliated Hospital of Xuzhou Medical University, Xuzhou, Jiangsu, 221002, China.; 2 Department of Epidemiology and Biostatistics, School of Public Health, Xuzhou Medical University, Xuzhou, Jiangsu, 221004, China.; 3 Center for Medical Statistics and Data Analysis, School of Public Health, Xuzhou Medical University, Xuzhou, Jiangsu, 221004, China.

**Keywords:** DLBCL, GNRI, PNI, Prognosis, elderly patients

## Abstract

**Background:** Geriatric nutritional risk index (GNRI) and prognostic nutritional index (PNI) are associated with prognosis of various malignancies. Although GNRI and PNI indicates prognosis in some clinical settings, the values of GNRI and PNI on the prognosis of geriatric patients with Diffuse Large B‐Cell Lymphoma (DLBCL) is unclear. This retrospective analysis aimed to explore the prognostic values of GNRI and PNI in elderly DLBCL patients.

**Methods:** A total of 133 geriatric patients with DLBCL were recruited from Affiliated Hospital of Xuzhou Medical University, and clinicopathological variables were analyzed. X-Tile program, restricted cubic spline (RCS) and time-dependent receiver operating characteristic (ROC) analysis were used to determine optimal cut-off points of GNRI, PNI and other continuous variables; univariate and multivariate Cox proportional hazards analyses were used for variables selection; Kaplan‐Meier curve was utilized to analyze the influence of variables on prognosis; log-rank test was performed for difference evaluation between groups.

**Results:** The optimal cut-off points for GNRI and PNI were 106.26 and 47 by using RCS. Multivariate analysis showed that PNI, age, hemoglobin, liver invasion and central nervous system invasion were independent prognostic factors for elderly patients with DLBCL, and PNI was (*P* = 0.001, *HR* = 0.413, 95% *CI* (0.240-0.710) a stronger predictor. Low PNI could predict worse prognosis independently of elderly patients of DLBCL and could re-stratify patients in GCB group, CD5 positive group BCL-2 positive group, and BCL-6 positive group.

**Conclusions:** PNI was an independent adverse factor for elderly DLBCL and patients with low PNI in GCB group, CD5 positive group and BCL-6 positive group were with poor survival.

## Introduction

Diffuse large B-cell lymphoma (DLBCL) is a prevalent subtype of lymphoma and is highly heterogeneous in gene-based molecular stratification, cell-of-origin, immune markers and prognosis, accounting for about 40% of non-Hodgkin lymphoma (NHL) 1. In the era of rituximab-based immunochemotherapy, 40% patients will develop relapse or refractory 2. Due to complex molecular genetic characteristics, poor nutritional status and frail conditions 3, 4, few of geriatric patients are suit for intensive chemotherapy and hematopoietic stem cell transplantation. Clinical factors based prognostic systems, such as International Prognostic Index (IPI), NCCN-IPI, and GELTAMO-IPI 5-7 were used for the risk stratification of patients with DLBCL. However, these indices did not take the nutritional status of patients into account.

The nutritional status is a key factor affecting the response and prognosis of cancer patients, and about 30%-40% of patients suffer from malnutrition. Malnutrition refers to varying degrees of overnutrition or undernutrition according to the definition of American Society for Parenteral and Enteral Nutrition (ASPEN). Malnutrition can lead to many adverse clinical outcomes, including the increase of complications, morbidity, mortality, and the reduction in patient survival 8. The aging process causes a loss of bone density and increases the risk of osteoporosis. In addition, changes in the digestive system and sensory system in elderly patients will result in a reduction in the gut motility and inappetence 9, 10. A previous research suggested that about 50% of deaths could be attributed to malnutrition in tumors 11. Mansoor R et al. also showed that the risk of acute mortality increased with decreasing nutrition status in mature B-cell NHL 12. There are several highly sensitive nutritional screening tools for further diagnosis of malnutrition and appropriate interventions, such as nutritional risk index (NRI), and prognostic nutritional index (PNI) 13. Furthermore, geriatric nutrition risk index (GNRI), a modified version of the developed NRI, is specifically for the elderly population 14. Previous studies have shown that GNRI was a prognostic factor for esophageal, gastric, chronic kidney diseases and DLBCL 15-18. A meta-analysis indicated that low PNI may be interpreted as adverse prognosis for DLBCL patients 19. However, few studies have explored the effects of PNI and GNRI on the prognosis of elderly patients with DLBCL. This retrospective study aimed to evaluate the prognostic significance of GNRI and PNI in geriatric patients with DLBCL in the rituximab era.

## Materials and methods

### Patients

We carried out a retrospective study of 133 newly diagnosed geriatric patients with DLBCL (≥60 years) in Affiliated Hospital of Xuzhou Medical University from January 2014 to May 2018. All patients included in this study were with pathological diagnosis of DLBCL and treated with rituximab based immunochemotherapy (R-CHOP and R-CHOP like with median treatment line of 2 (1-4)). Exclusion criteria: 1) patients with other hematological malignancies; 2) patients with special types of lymphoma (primary central nervous system lymphoma, primary mediastinal DLBCL, transformed DLBCL).

The collected information of initial diagnosis as DLBCL included: gender, age, BMI, extranodal involvement, performance status 20, presence of bulky disease (≥7.5 cm), B symptoms, ferritin, lactate dehydrogenase (LDH), albumin, stage, neutrophils (Neu), White blood cell count (WBC), Red blood cell count (RBC), hemoglobin, cell of origin, and immunological markers. Follow-up was conducted through reviewing in-patient medical records and making phone calls. Overall survival (OS) was calculated as the interval between the time of diagnosis and death from any cause or the last follow-up.

### Assessment of nutritional status

GNRI is an accurate nutrition-related risk index tool and is calculated from albumin and BMI using the formula 14:

*GNRI* = [1.489 × *albumin (g/L)*] + [41.7 × (*weight/WLo*)]

where: WLo is the ideal weight, which was calculated by the following formula:

*For men: H* - 100 - [(*H* - 150)/4]

*For women: H* - 100 - [(*H* - 150)/2.5]

where: *H* is the height.

Based on the values of GNRI, four grades of nutrition-related risk were defined: major risk (GNRI: < 82), moderate risk (GNRI: 82-92), low risk (GNRI: 92-98), and no risk (GNRI: > 98).

PNI, based on serum albumin and lymphocytes, is a scoring system that reflects the nutritional status and immune status of patients. It is calculated from using the formula^13^:

*PNI* = 10 × *albumin (g/dL)* + 0.005 × *lymphocyte count (/mm^3^)*

### Statistical analysis

Baseline clinical characteristics were described by variable type using median and interquartile range. Outliers were verified by the hospital medical record system. All cases had complete clinical information to avoid unnecessary bias. Continuous variables were transformed into categorical variables by X-Tile program (Yale University, New Haven, CT, USA) 21, restricted cubic spline (RCS) 22 and time-dependent receiver operating characteristic (ROC) curve analysis. Kaplan-Meier analysis was used to explore the effect of pathological factors combined with PNI and GNRI on survival, and log-rank test was performed for the difference between groups. Cox proportional hazard model was used to analyze the univariate association between clinical features and prognosis. All variables with *P<*0.1 in univariable analysis were included in the multivariable model. Multivariate Cox analysis was performed to identify the predictive prognostic variables. The best prediction variable set was obtained by forward stepwise regression and Akaike Information Criteria (AIC) 23 was used to evaluate the optimal model. *P-*values < 0.05 (two‐tailed) were considered as significant. Statistical analysis was conducted with IBM SPSS version 19.0 for Windows software program (IBM Corp, Armonk, NY, USA) and R software (version 4.0.3; http://www.Rproject.org).

## Results

### Clinical Characteristics

The follow-up deadline was May 1, 2020. The median follow-up time was 35.2 months (95%* CI:* 29.296-41.104) and the median OS was 19.4 months (95%* CI:* 11.490-27.310). At the end of follow-up, a total of 75 (56.3%) deaths occurred. Median age at diagnosis was 71 years (range: 60-91), of whom 67 (50.3%) were males. Ann Arbor stage I/II and III/IV accounted for 59.4% and 40.6%. 12.1% of patients had B symptoms. Patient characteristics were shown in Table 1.

### Cut-off points identification of GNRI, PNI and clinical continuous variables

**Figure 1:**
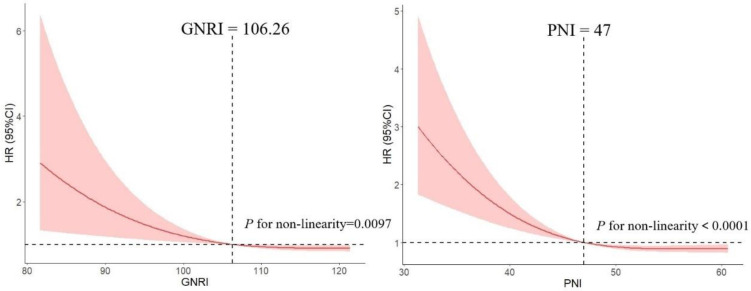
Association between GNRI, PNI and the risk of DLBCL by using restricted cubic spline (RCS), allowing for nonlinear effects.

We used RCS model with 3 knots to simulate the relationship between GNRI and the risk for DLBCL. Significant nonlinear dose-response association was showed in the relationship between GNRI and the risk (*P* = 0.0097). And dose-response relationship analysis showed that with the continuous change of GNRI, the association strength of risk decreased nonlinearly. Similarly, we used this method to calculate the optimal cut-off point of PNI (Figure 1).

**Figure 2:**
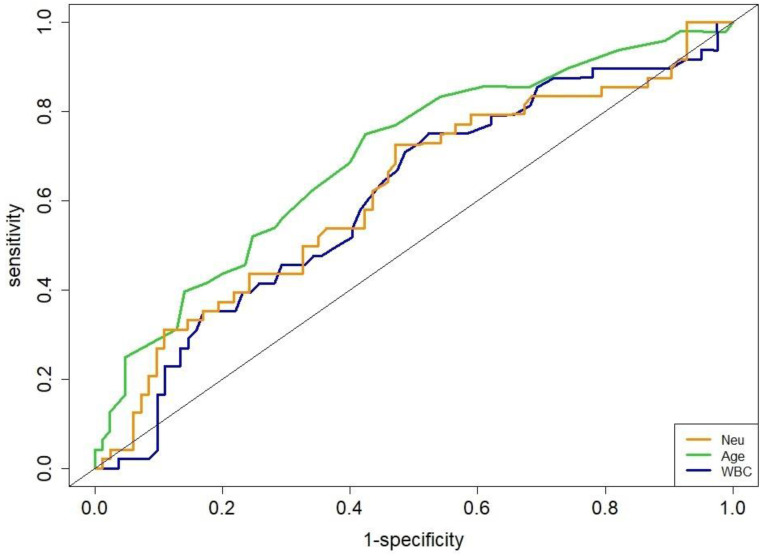
Receiver operating characteristic curves for age, WBC and Neu.

The area under the ROC (AUROC) curve for age was 0.70 to obtain an optimal cut-off value of 69-y (sensitivity, 75.7%; specificity, 62.5%; *P* = 0.004). The WBC had an AUROC of 0.66 for an optimal cut-off value of 5.8×10^9^/L (sensitivity, 70.3%; specificity, 51.8%;* P* = 0.042). The Neu had an AUROC of 0.67 for an optimal cut-off value of 3.84×10^9^/L (sensitivity, 68.0%; specificity, 58.9%;* P* = 0.051, Figure 2).

**Figure 3:**
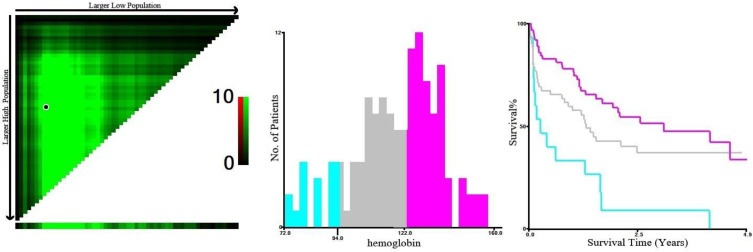
X-Tile analysis of survival data based on DLBCL patients reveals a continuous distribution based on hemoglobin. The plots show the *χ^2^* log-rank values produced when dividing the patients with two cut-points, producing high, middle, and low subsets.

Based on X-Tile program, the maximum chi-squared points 14.102 and 6.856 were reached when the values of 94 g/L and 122 g/L were used as the optimal cut-off points of hemoglobin. According to the hemoglobin distribution at diagnosis, patients were divided into three groups: <94 g/L, 94-122 g/L, and ≥122 g/L (*P* < 0.05, Figure 3).

### Univariate and multivariate analysis of elderly patients with DLBCL

The results of univariate analysis found that PNI, hemoglobin, age, LDH, neutrophils, liver invasion, central nervous system invasion, β2-microglobulin, GNRI, BMI, BCL-2 and WBC significantly affected OS (*P* < 0.05, Table 2). PNI and hemoglobin appeared to be stronger predictors (*P* < 0.001). After iterative analysis of the multivariate model, the final prognostic indicator was composed of five adverse factors (Table 2). Nevertheless, LDH in the multivariate model was not observed to be predictable (*P* = 0.289, *HR* = 1.475, 95% *CI:* 0.718-3.030).

### Prognostic values of PNI and GNRI among elderly patients with DLBCL

**Figure 4:**
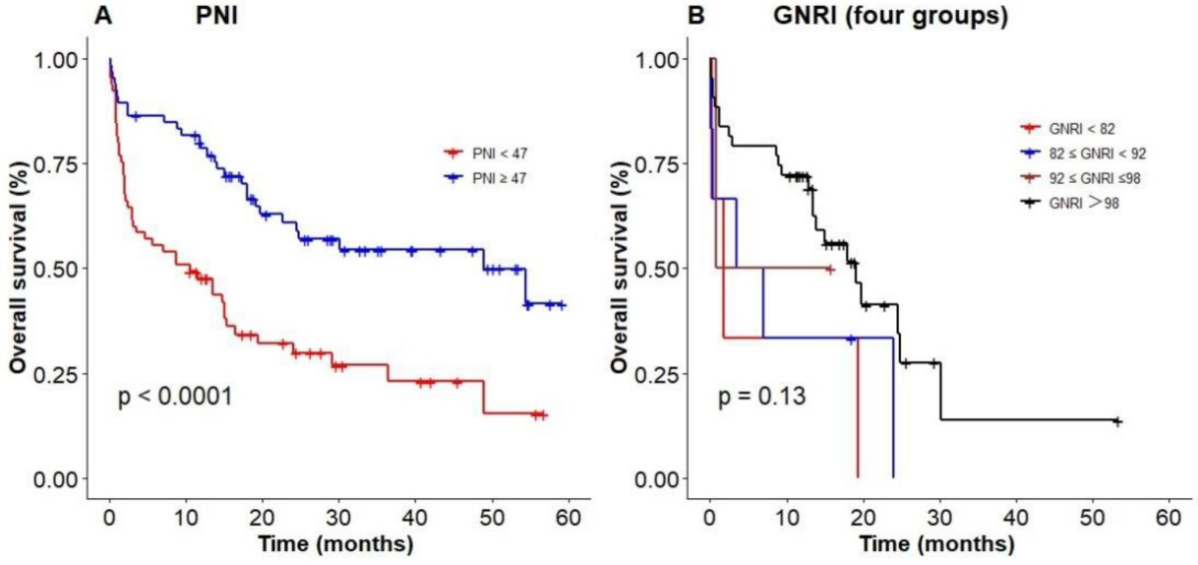
Poor nutritional status predicting an inferior survival based on (A) PNI, (B) GNRI (four groups).

**Figure 5:**
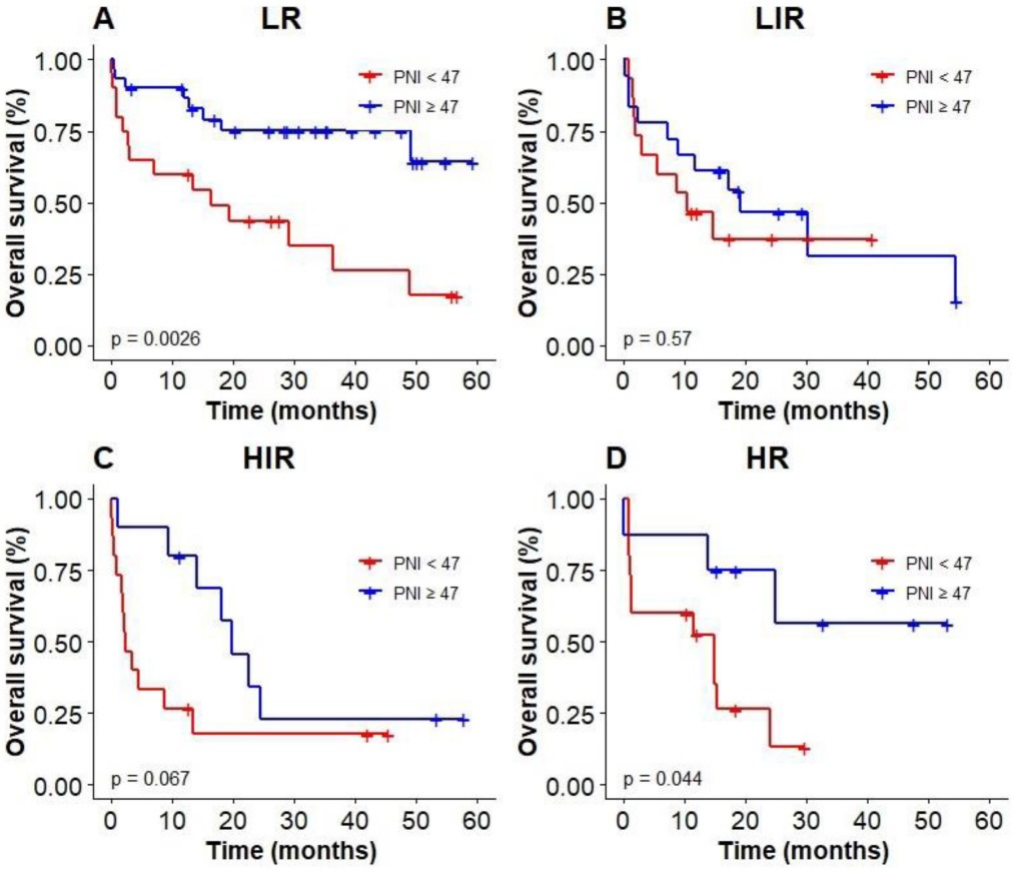
Kaplan-Meier survival analysis of OS based on PNI of elderly patients with DLBCL in different IPI groups.

We used the optimal cut-off values calculated by RCS to evaluate the prognosis among elderly patients with DLBCL. The Kaplan‐Meier results showed patients with low PNI were associated with poor OS (*P* < 0.0001, with the 3-year OS of 23% vs 52%, Figure 4A), compared with high PNI patients with DLBCL. However, patients could not be accurately stratified by using the recommended cut-off values of GNRI (*P* = 0.13, Figure 4B). In addition, PNI successfully differentiated patients with poor prognosis in low-risk group and high-risk group (3-year OS 26.2% vs 69.3%, *P* = 0.003; 3-year OS 0 vs 55.2%, *P* = 0.044; Figure 5A, D). However, PNI could not accurately stratify patients in other groups of IPI scoring system (Figure 5B, C).

**Figure 6:**
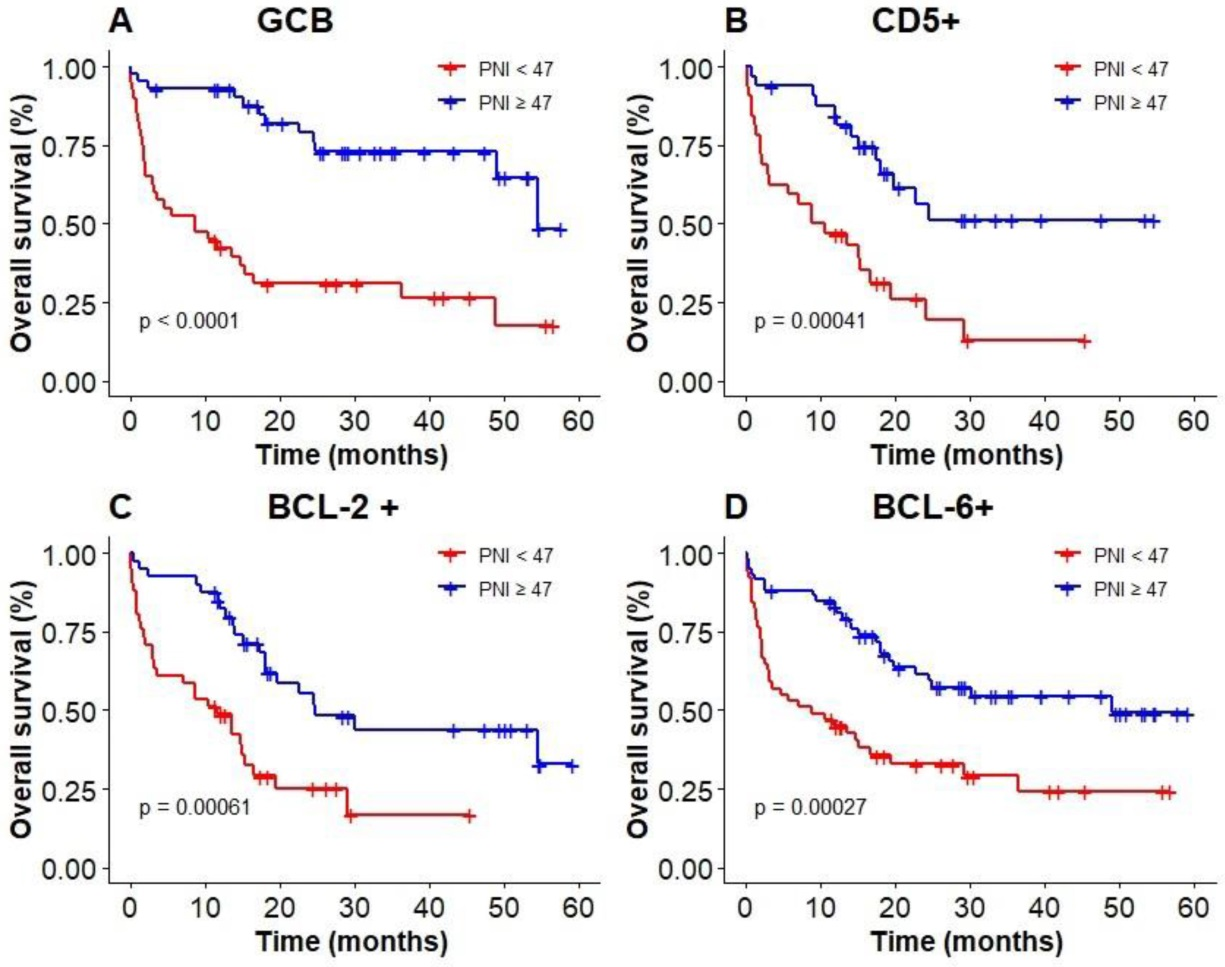
Kaplan‐Meier survival curves of PNI in elderly DLBCL patients among different subgroups; (A): GCB; (B): CD5+; (C): BCL-2+; (D): BCL-6+.

The results of subgroup analysis showed that a significant deterioration of OS in GCB, CD5 positive, and BCL-6 positive groups with malnutrition identified by PNI (Figure 6A, B, D). OS was significantly worsened by PNI in BCL-2 positive group (Figure 6C). PNI could not accurately distinguish patients among BCL-6 negative group and non-GCB group.

**Figure 7:**
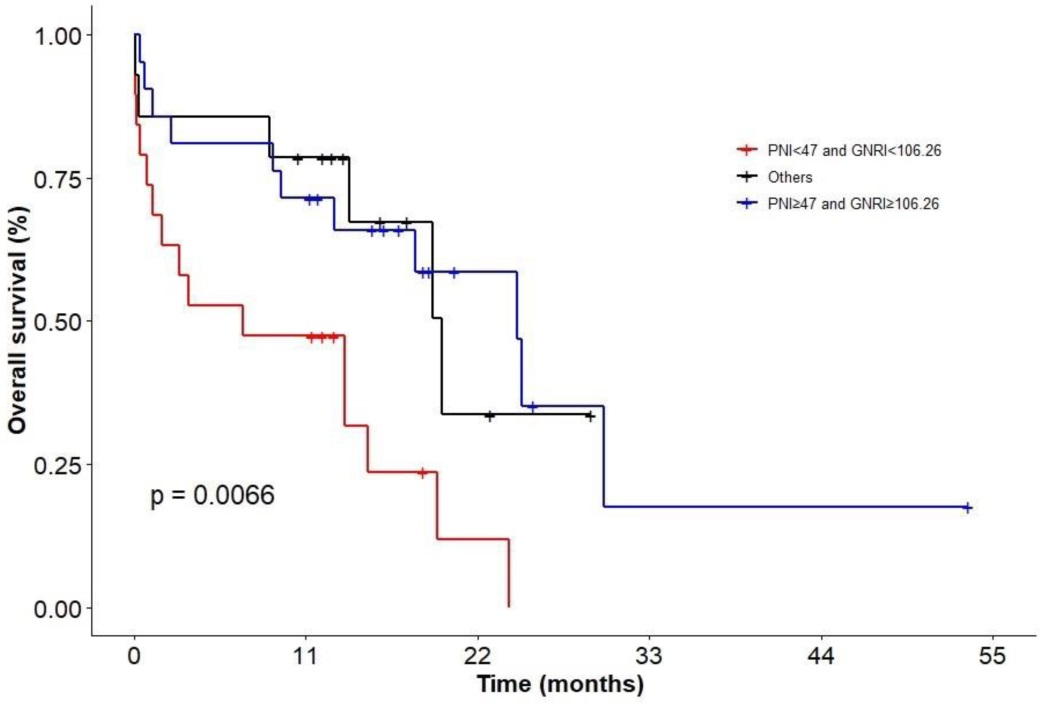
KM analysis of patients in different nutritional status groups.

In addition, we evaluated patients with combination of PNI and GNRI. KM analysis showed that patients in status with PNI < 47 and GNRI < 106.26 had the worst survival (*P* = 0.0066; Figure 7; 2-y OS: 0 vs 33.7% vs 58.6%; 3-y OS: 0 vs 0 vs 17.6%), but there was no difference between the group of patients with PNI ≥ 47 and GNRI ≥ 106.26 and other groups.

## Discussion

Malnutrition is frequent in patients with tumors and is associated with the occurrence and progression. In addition, advanced age patients are thought to be more prone to nutritional problems, leading to inadequate treatment and poor survival 24-26. This retrospective study suggested that PNI could be used as an independent prognostic factor for elderly patients with DLBCL. In addition, patients with low PNI in GCB group, CD5 positive group and BCL-6 positive group were with poor survival.

GNRI and PNI are common indicators to evaluate the nutritional status of patients. Several studies have confirmed that GNRI could be used as a prognostic factor in DLBCL patients 18, 27, 28. In this study, we calculated the optimal cut-off value of GNRI by RCS, which could be used to accurate stratify elderly patients. Univariate analysis results revealed that GNRI had an impact on the prognosis of elderly patients with DLBCL, and patients with high levels of GNRI were with a longer survival. However, GNRI was not an independent prognostic factor for OS in multivariate analysis in our study which was consistent with the results of a previous domestic study 29.

Our study also showed that PNI was suitable for assessing the nutritional status of elderly patients with DLBCL. Multivariate analysis showed that PNI was a strong prognostic predictor. The relationship between nutritional status and prognosis of patients could be accurately interpretated, and precise stratification of patients in the low-risk and high-risk groups could be achieved by using IPI system. In the following subgroup analysis, we also found that in BCL-6 positive group, BCL-2 positive group, CD5 positive group and GCB group, patients with high PNI had a significantly higher OS than that of patients with low PNI.

Low level of albumin is associated with poor prognosis in cancer patients and may serve as an independent indicator for requiring aggressive nutritional interventions 30, 31. Previous studies have shown that lymphocyte count is a prognostic factor for adverse event rates and clinical outcomes in patients with DLBCL 32, 33. It was worth noting that in this study univariate analysis showed that albumin and lymphocyte count had no effect on prognosis of DLBCL, whereas the PNI based on the two indicators was an independent prognostic factor. It suggested that PNI could be used to evaluate the nutritional status of patients in subsequent clinical practice. In addition, we evaluated patients with combination of PNI and GNRI and the results showed that patients in status with PNI < 47 and GNRI < 106.26 had the worst survival.

In conclusion, nutritional status has an impact on the survival of elderly DLBCL patients. The results suggest that PNI can be used as an independent prognostic indictor of elderly patients with DLBCL, and PNI can predict worse prognosis of patients in GCB group, CD5 positive group, BCL-2 positive and BCL-6 positive group.

## Figures and Tables

**Table 1:** Basic clinical information of elderly patients with DLBCL

Characteristics	n (%)
**Gender**	
male	67 (50.4)
female	66 (49.6)
**ECOG PS score**	
0-1	105 (78.9)
≥2	28 (21.1)
**Ann Arbor stage**	
I-II	79 (59.4)
III-IV	54 (40.6)
**Bulky**	
absence	122 (91.7)
presence	11 (8.3)
**B symptoms**	
absence	117 (87.9)
presence	16 (12.1)
**LDH**	
normal	75 (56.4)
elevated	58 (43.6)
**Liver invasion**	
no	125 (93.9)
yes	8 (6.1)
**CNS invasion**	
no	113 (84.9)
yes	20 (15.1)
**Bone marrow invasion**	
no	105 (78.9)
yes	28 (21.1)
**IPI score**	
LR/LIR	85 (63.9)
HIR/HR	48(36.1)
**ASCT**	
no	128 (96.2)
yes	5 (3.8)
**GNRI**	
<82	15 (11.3)
82-92	26 (19.5)
92-98	28 (21.1)
>98	64 (48.1)

Note: DLBCL: Diffuse Large B‐Cell Lymphoma; ECOG PS score: Eastern Cooperative Oncology Group performance status score; LDH: lactic dehydrogenase; CNS invasion: Central nervous system invasion; IPI: International Prognostic Index; LR: low risk; LIR: low intermediate risk; HIR: high intermediate risk; HR: high risk; ASCT: Autologous stem cell transplantation.

**Table 2:** Univariate and multivariate analyses of prognostic factors for OS

Univariate analysis				Multivariate analysis			
Variables	*HR*	95% *CI*	*P*	Variables	*HR*	95% CI	*P*
PNI (≥47 vs. <47)	0.394	(0.246-0.632)	<0.001	PNI (≥47 vs. <47)	0.413	(0.270-0.710)	0.001
**Hemoglobin**				Hemoglobin			
<94	1			<94	1		
94-122	0.46	(0.240-0.882)	0.019	94-122	0.35	(0.119-0.208)	0.046
≥122	0.296	(0.153-0.573)	<0.001	≥122	0.673	(0.474-0.956)	0.027
Age (≥69 vs. <69)	1.054	(1.022-1.088)	0.001	Age (≥69 vs. <69)	1.986	(1.167-3.380)	0.011
LDH (ratio >1 vs. ≤1)	1.973	(1.246-3.123)	0.004	Liver invasion (yes vs. no)	4.868	(1.825-12.988)	0.002
Neutrophils (≥3.84 vs. <3.84)	1.948	(1.199-3.167)	0.007	CNS invasion (yes vs. no)	2.543	(1.218-5.310)	0.013
IPI (HIR/HR vs.LR/LIR)	1.299	(1.071-1.576)	0.008	WBC (≥5.8 vs. <5.8)	2.033	(0.853-4.847)	0.109
β2-MG (elevated vs. normal)	2.315	(1.169-4.586)	0.016				
Liver invasion (yes vs. no)	3.08	(1.329-7.136)	0.009				
GNRI (≥106.26 vs. <106.26)	0.675	(0.482-0.944)	0.022				
CNS invasion (yes vs. no)	1.936	(1.097-3.416)	0.023				
WBC (≥5.8 vs. <5.8)	1.81	(1.098-2.983)	0.02				
BMI (<24 vs. ≥24)	0.871	(0.773-0.982)	0.024				
BCL-2 (positive vs. negative)	2.037	(0.096-0.317)	0.043				
BM involvement (yes vs. no)	1.385	(0.600-3.198)	0.445				

Note: PNI: prognostic nutritional index; GNRI: geriatric nutritional risk index; IPI: International Prognostic Index; HIR: high intermediate risk; HR: high risk; LR: low risk; LIR: low intermediate risk; WBC: White blood cell count; β2-MG: β2-microglobulin; CNS invasion: Central nervous system invasion. BM involvement: Bone marrow involvement.
